# Blood meal identification in the cryptic species *Anopheles gambiae and Anopheles coluzzii* using MALDI-TOF MS

**DOI:** 10.1051/parasite/2018041

**Published:** 2018-07-27

**Authors:** Fatalmoudou Tandina, Maureen Laroche, Bernard Davoust, Ogobara K Doumbo, Philippe Parola

**Affiliations:** 1 Aix Marseille Univ, IRD, AP-HM, SSA, VITROME, IHU-Méditerranée Infection Marseille France; 2 Department of Epidemiology of Parasitic Diseases, Malaria Research and Training Center, University of Science, Techniques and Technologies of Bamako Bamako Mali; 3 Aix Marseille Univ, IRD, APHM, MEPHI, IHU-Méditerranée Infection Marseille France

**Keywords:** Blood meal identification, MALDI-TOF MS, *Anopheles coluzzii*, *Anopheles gambiae*

## Abstract

Matrix-assisted laser desorption/ionization time-of-flight mass spectrometry (MALDI-TOF MS) has recently emerged in entomology as a technique to identify arthropods and their blood meal source. In this study, female *Anopheles gambiae* were fed on five host blood sources: ocelot (*Leopardus pardalis*), binturong (*Arctictis binturong)*, springbok (*Antidorcas marsupialis)*, jaguar (*Panthera onca)* and Hamadryas baboon (*Papio hamadryas)*, while *Anopheles coluzzii* were fed on three hosts: dromedary (*Camelus dromedarius)*, Barbary sheep (*Ammotragus lervia)* and pig (*Sus scrofa)*. We obtained the MS spectra from 240 engorged mosquito abdomens and selected high quality ones from 72 mosquito abdomens to upgrade our home-made database. We excluded from the analysis any spectra of low quality (*n* = 80), and the remaining 88 specimens were subjected to a blind test analysis against the home-made database. We obtained 100% correct identification of the blood meal source for the specimens collected, 1, 12 and 24 h post-feeding, whereas for the specimens collected 36 h post-feeding, the correct identification rate decreased dramatically. We confirm here that MALDI-TOF MS can be used to identify the blood meal origin of freshly engorged mosquitoes, which opens new perspectives for further studies, including the impact of the mosquito species on blood meal identification.

## Abstract (fr)

L’identification par spectrométrie de masse à temps de vol par désorption/ionisation assistée par matrice (MALDI-TOF MS) a récemment émergé en entomologie pour l’identification des arthropodes et de leur source de sang. Des femelles d’*Anopheles gambiae* ont été nourries de sang de cinq hôtes, ocelot (*Leopardus pardalis*), binturong (*Arctictis binturong*), springbok (*Antidorcas marsupialis*), jaguar (*Panthera onca*) et babouin Hamadryas (*Papio hamadryas*), et des femelles d’*Anopheles coluzzii* ont été nourries sur trois hôtes, dromadaire (*Camelus dromedarius*), mouflon à manchettes (*Ammotragus lervia*) et porc (*Sus scrofa*). Nous avons obtenu les spectres MS à partir de 240 abdomens de moustiques engorgés et avons sélectionné ceux de 72 abdomens de moustiques de haute qualité pour améliorer notre base de données maison. Nous avons exclu de l’analyse les spectres de faible qualité (*n* = 80) et les 88 échantillons restants ont été soumis à une analyse de test en aveugle contre la base de données maison. Nous avons obtenu 100 % d’identification correcte de la source de sang pour les échantillons collectés, 1, 12 et 24 heures après l’alimentation, mais le taux d’identification correct a diminué de façon spectaculaire pour les échantillons collectés 36 heures après l’alimentation. Nous confirmons ici que la MALDI-TOF MS peut être utilisée pour identifier l’origine des repas sanguins des moustiques fraîchement engorgés et ouvre de nouvelles perspectives pour d’autres études, y compris l’impact des espèces de moustiques sur l’identification des repas sanguins.

## Introduction

The analysis and identification of mosquito blood meals is essential to the study of vector bite behavior (anthropophilic or zoophilic). Several methods have been developed to identify the vertebrate host of mosquito blood meals, including serological tools such as precipitin and ELISA tests [3, 4]. Although these methods provide valuable information, they present several drawbacks, including the availability of specific antisera against antibodies, and their cross-reactivity [3, 4]. A molecular biology approach has been adopted as an effective strategy to identify mosquito blood sources [3, 4]. Nevertheless, DNA sequencing can be costly and time consuming [3, 4].

The matrix-assisted laser desorption/ionization time-of-flight mass spectrometry (MALDI-TOF MS) process is based on acidic extraction of proteins from an organism of interest, which are co-crystallized with a matrix. The molecules are ionized and propelled in a flight tube according to their mass-to-charge ratio. The detection of each molecule will generate an individual peak, therefore providing an overall spectrum which will be highly specific to the sample [22]. In recent years, this process has revolutionized clinical microbiology [21]. Recently, MALDI-TOF MS has emerged as an innovative tool to identify arthropods [8, 24, 25]. Also, in preliminary reports, MALDI-TOF MS has appeared as a promising tool to identify mosquito blood meals, using mosquitoes experimentally engorged in the lab, as well as using engorged mosquito abdomens crushed on Whatman filter papers (WFPs) collected during entomological field surveys [16–18, 23]. In these studies, a home-made MALDI-TOF MS database was established with abdomen MS spectra from mosquitoes freshly engorged on several vertebrate hosts [17, 18]. Other recent studies have used a tandem mass spectrometry approach to identify tick and triatomine blood meals [6, 7, 10, 19] by the trypsin digestion of the samples [6, 7, 10, 11, 19].

The goal of this study was to confirm the discriminative power of the MALDI-TOF MS tool for mosquito blood meal identification using a large panel of host blood vertebrates. For this purpose, *Anopheles coluzzii* and *Anopheles gambiae* Giles mosquitoes, two cryptic species among the main vectors of malaria in Africa [1, 5], were artificially fed with different host species that were not previously included in the home-made database.

## Materials and methods

### Ethical statement

The *Sus scrofa* blood sample was collected by a veterinary specialist, with the agreement of the owners, in accordance with standards relating to animal welfare. All other animals were living in zoos and were first anesthetized by a veterinarian responsible for monitoring their health. This protocol was approved by the Animal Ethics Committee of Marseille (C2EA-14), and by the French authorities. The blood samples were processed and stored in accordance with the World Health Organization’s Good Clinical Laboratory Practice guidelines and documents on blood sample handling procedures. (Good Clinical Laboratory Practice, 2009). Mosquitoes were reared using the International Conference on Harmonization/Good Laboratory Practices (ICH/GLP) procedures.

### Laboratory rearing of *Anopheles gambiae* Giles and *Anopheles coluzzii*

**Figure 1.**
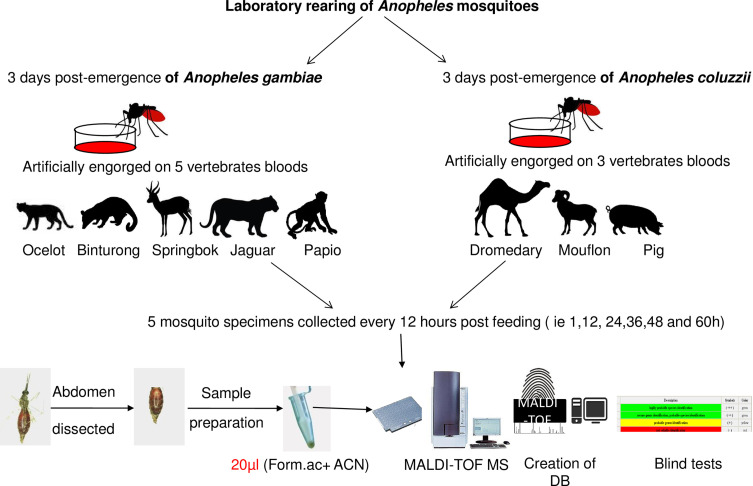
Experimental workflow for blood meal identification using MALDI-TOF MS (Form.ac: Formic acid ; ACN: acetonitrile ; DB: database).

Both mosquito species were reared using standard methods at a temperature of 26 ± 1 °C, relative humidity of 70–90%, and over a photoperiod of 12 h (light/dark). *Anopheles coluzzii* adult females were artificially fed through a Parafilm-membrane on three blood vertebrates, namely *Camelus dromedarius*, *Ammotragus lervia* and *Sus scrofa*. The *An. gambiae* Giles females were artificially engorged on five vertebrate blood sources, namely *Leopardus pardalis*, *Arctictis binturong*, *Antidorcas marsupialus*, *Panthera onca* and *Papio hamadryas* for 2 h, as previously described [17, 18]. Engorged females were transferred to a new cage and fed with 10% glucose solution. Five engorged females were harvested between 1 and 60 h post-blood feeding, every 12 h (i.e. 1, 12, 24, 36, 48, and 60 h). The mosquito abdomens were placed in individually labeled vials. A flowchart illustrating the main steps is presented in Figure 1.

### Spectral analysis

The individual mosquito abdomens were manually crushed and prepared as described previously [17]. MALDI-TOF MS spectra from engorged mosquito specimens were evaluated by analyzing the average spectra obtained from the four spectra of each sample tested using Flex Analysis 3.3 and ClinPro-Tools 2.2 software. These four spectra correspond to the four spots of the same sample on the MALDI-TOF target plate. High quality spectra (*n* = 40) were selected to create a dendrogram. Dendrograms are based on the results of a Composite Correlation Index (CCI) matrix. The CCIs are calculated by dividing spectra into intervals and comparing these intervals across a dataset. The composition of correlations of all intervals provides the CCI, which is used as a parameter that defines the distance between spectra [9].

### Database upgrading and blind tests

In order to upgrade the MS arthropod home-made database (Additional file: Table S1), the four high quality spectra from at least three specimens per species, that were fed on the same blood and harvested at the same time point, were combined by the automated function of MALDI-Biotyper software v.3.3 to create 72 reference spectra. Spectra of low quality were excluded from the analysis. Subsequently, a blind test against the updated database was performed with all remaining MS spectra from the abdominals of clogged mosquitoes on eight separate host bloods. The results of the database queries are presented as Log Score Values (LSVs) for each spectrum given corresponding to a matched degree of signal intensities of mass spectra of the query and the reference spectra. LSVs ranged from 0 to 3 [9]. LSVs greater than 1.8 were considered as the threshold value for relevant identification, as previously published [17].

## Results

A total of 240 MS spectra from 150 *Anopheles gambiae* Giles and 90 *Anopheles coluzzii* engorged abdomens, collected from 1 to 60 h post-blood feeding and spotted in quadruplicate, were obtained.

**Figure 2.**
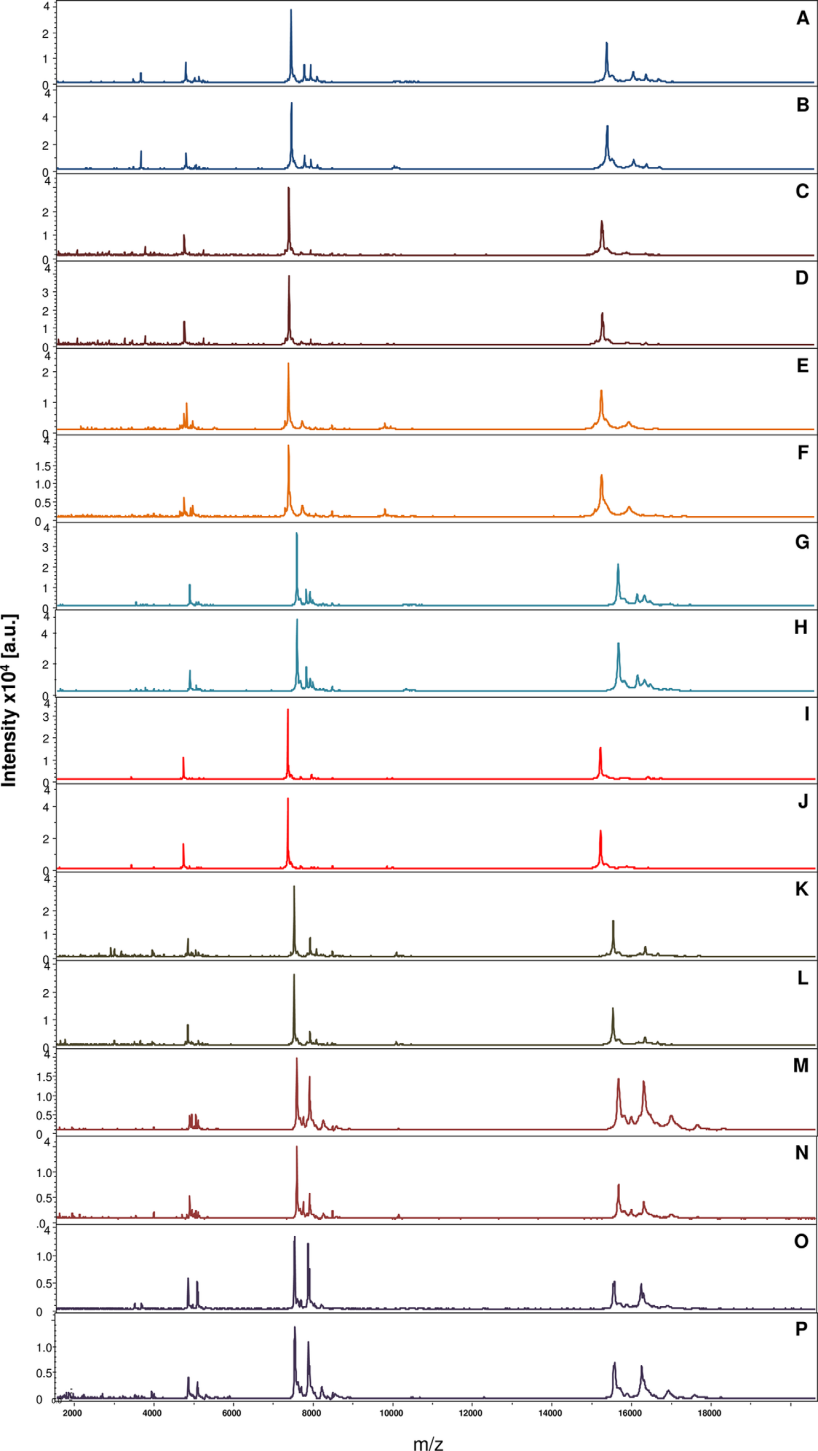
MALDI-TOF MS spectra of *Anopheles gambiae* Giles and *Anopheles coluzzii* abdomen protein extracts engorged on host blood vertebrates. All mosquitoes were collected only at times 1 and 24 h post-feeding. Alignment of MS spectra from: H1_*An_coluzzii*_*Camelus dromedarius* (A), H24_*An_coluzzii*_ *Camelus dromedarius* (B), H1_ *An_coluzzii Ammotragus lervia* (C), H24_ *An_coluzzii Ammotragus lervia*(D), H1_ *An_coluzzii* _*Sus scrofa* (E), H24_ *An_coluzzii* _*Sus scrofa* (F), H1_ *An_gambiae* _*Papio hamadryas* (G), H24_ *An_gambiae* _*Papio hamadryas* (H), H1_*An_gambiae*_ *Antidorcas marsupialus* (I), H24_*An_gambiae*_ *Antidorcas marsupialus* (J), H1_*An_gambiae*_*Arctictis binturong* (K), H24_*An_gambiae*_ *Arctictis binturong* (L), H1_ *An_gambiae*_ _*Panthera onca* (M), H24_ *An_gambiae*_ _*Panthera onca* (N), H1_*An_gambiae*_*Leopardus pardalis* (O), and H24_*An_gambiae*_ *Leopardus pardalis* (P). a.u. arbitrary units; *m*/*z* mass-to-charge ratio.

Comparison of these MS spectra revealed high reproducibility of protein profiles between the same vertebrate blood samples from mosquito abdomen protein extracts using Flex Analysis software (Fig. 2). Among the 240 spectra, 72 spectra of the highest quality were used to upgrade the home-made database. This database previously contained reference spectra for several arthropods, including reference spectra derived from the legs of 50 mosquito species, as well as reference spectra of *Anopheles gambiae* abdomens engorged on 17 different hosts (Table S1).

We excluded from the analysis the spectra of low quality (*n* = 80) collected from 48 to 60 h post-blood feeding and the remaining MS spectra from 88 mosquito abdomens collected from 1, 12, 24 and 36 h, engorged on eight blood vertebrates, were tested against the home-made database. The specimens (*n* = 48) collected 1, 12 and 24 h post-feeding, revealed 100% correct identification of the host blood origin. The log score values (LSVs) for these freshly engorged specimens ranged from 1.912 to 2.918 (Table 1). Regarding the specimens collected at 36 h post-feeding (*n* = 40), the percentage of correct identification (with LSVs ranging from 1.396 to 2.207) dropped to less than 10%. Among them, 4/40 *Anopheles coluzzii* abdomens engorged on *Sus scrofa* blood (*n* = 2) and *Ammotragus lervia* blood (*n* = 2) were correctly identified with an LSV ranging from 1.800 to 2.207. For the remaining specimens collected at 36 h (*n* = 36, 90%), an incorrect blood source was identified (Table 1). As for the specimens collected 48 and 60 h post-feeding (*n* = 80), all MS spectra were of low quality.

**Table 1.** Mosquito engorged abdomen spectra used for the home-made database and identification according to post-feeding period.

Mosquito species	Blood meal source	Time of collection (hours)	Number of specimens used to upgrade the DB	Number of specimens used for blind tests	LSVs obtained from blind tests against DB	Mean of range	Blood meal identified by MS
*Anopheles coluzzii*	*Ammotragus lervia*	1–24	9	6	[2.518–2.918]	2.756	*Ammotragus lervia*
*Anopheles coluzzii*	*Ammotragus lervia*	36	0	5	[1.789–2.011]	1.873	*/*
*Anopheles coluzzii*	*Camelus dromedarius*	1–24	9	6	[2.418–2.830]	2.706	*Camelus dromedarius*
*Anopheles coluzzii*	*Camelus dromedarius*	36	0	5	[1. 597–2.029]	1.764	*/*
*Anopheles coluzzii*	*Sus scrofa*	1–24	9	6	[2.112–2.711]	2.615	*Sus scrofa*
*Anopheles coluzzii*	*Sus scrofa*	36	0	5	[1. 397–2.207]	1.905	*/*
*Anopheles gambiae* Giles	*Antidorcas marsupialus*	1–24	9	6	[2.530–2.807]	2.613	*Antidorcas marsupialus*
*Anopheles gambiae* Giles	*Antidorcas marsupialus*	36	0	5	[1. 562–1.941]	1.764	*/*
*Anopheles gambiae* Giles	*Arctictis binturong*	1–24	9	6	[2.585–2.892]	2.754	*Arctictis binturong*
*Anopheles gambiae* Giles	*Arctictis binturong*	36	0	5	[1.746 –1.892]	1.818	*/*
*Anopheles gambiae* Giles	*Leopardus pardalis*	1–24	9	6	[2.703–2.860]	2.773	*Leopardus pardalis*
*Anopheles gambiae* Giles	*Leopardus pardalis*	36	0	5	[1.747 –1.891]	1.818	*/*
*Anopheles gambiae* Giles	*Papio hamadryas*	1–24	9	6	[2.640–2.853]	2.756	*Papio hamadryas*
*Anopheles gambiae* Giles	*Papio hamadryas*	36	0	5	[1.652 –2.278]	1.998	*/*
*Anopheles gambiae* Giles	*Panthera onca*	1–24	9	6	[2.316–2.602]	2.521	*Panthera onca*
*Anopheles gambiae* Giles	*Panthera onca*	36	0	5	[1. 268–1.395]	1.342	*/*
Total			72	88			

Abbreviation: DB, home-made database.

**Figure 3.**
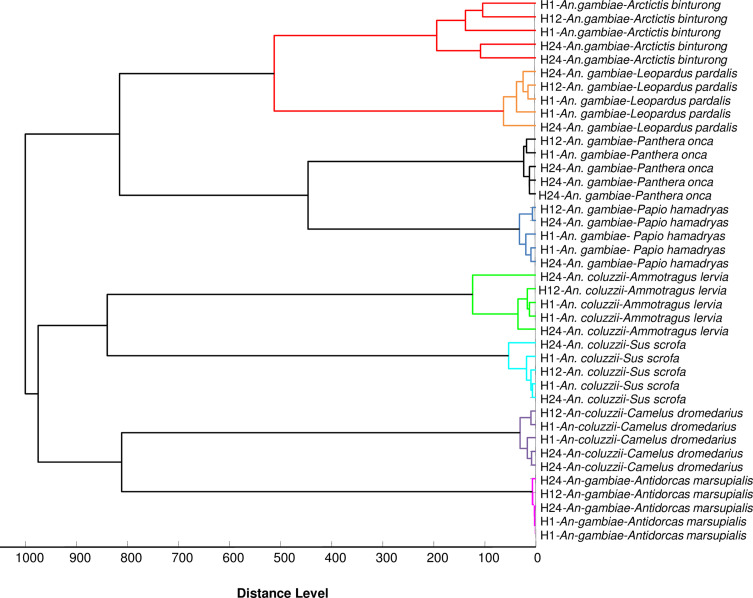
MSP dendrogram of MALDI-TOF MS spectra from *Anopheles gambiae* Giles and *Anopheles coluzzii* abdomens collected 1, 12 and 24 h post-feeding. MS spectra from two specimens from 1 to 24 h post-feeding and one specimen from 12 h post-feeding are represented. Blood meal host origins are indicated in the graph. Cluster analysis was performed by MALDI-Biotyper software v.3.3.

Cluster analysis revealed grouping on separate mosquito branches according to blood origin (Fig. 3). The MSP dendrogram showed that the spectra obtained from the abdomen of *An. gambiae* Giles, engorged on different felids (*Leopardus pardalis* and *Panthera onca*), were close (Fig. 3). The MSP dendrogram also revealed that the spectra obtained from the abdomen of *Anopheles gambiae* Giles, fed on different carnivore bloods, were close (Fig. 3).

## Discussion and conclusions

Our results confirm that MALDI-TOF MS is a promising tool for mosquito blood meal identification. The MS spectra generated from the abdomen of *Anopheles gambiae* Giles and *Anopheles coluzzii* revealed specific and reproducible blood results up to 24 h after feeding. In contrast, specimens collected 36 hours post-feeding revealed only 10% correct identification of the host blood origin, as suggested by preliminary studies [17, 18].

Cluster analysis revealed that all specimens fed with the same blood were clustered in the same branch (Fig. 3). Moreover, MALDI-TOF MS generated distinct MS profiles from the abdomens of *Anopheles gambiae* Giles freshly engorged with bloods of closely related animals, highlighting the specificity of this tool.

On the basis of this study and previous reports [17, 18], the field application of MALDI-TOF MS blood meal identification requires freshly engorged specimens. However, this is not problematic since field mosquitoes are caught in houses in the morning and traps are collected early, less than 24 h after the last blood meal [22]. In addition, Sella’s score visually determines the time and stage of digestion of mosquito blood meals and may be used to choose the mosquitoes that would give spectra of quality [14].

The MALDI-TOF approach may appear limited by the cost of the device and database comprehensiveness. Indeed, the correct identification of blood meals depends strictly on the presence of reference spectra in the database. Specimens without corresponding species reference spectra in the database matched with low log score values (<1.8). This underlines the need to continue database expansion with MS spectra from new vertebrate hosts [18]. However, reference spectra can be shared, avoiding the difficulties of creating a database. Nevertheless, when the MALDI-TOF device is purchased for clinical microbiology purposes, it can also be used for medical entomology at no additional cost [20].

This study opens new perspectives and further research is needed to better determine the usefulness of MALDI-TOF MS in medical entomology, and in particular for blood meal identification. In this study, the two included mosquito species, *Anopheles gambiae* Giles and *Anopheles coluzzii* were fed on different vertebrate bloods. However, *An. gambiae* and *An. coluzzi* are cryptic species, and it is not known at this stage whether similar results would be obtained by feeding mosquitoes of different genera. Although not noticeable for closely related species, the mosquito proteome might influence the obtained spectra and affect the resulting blood meal identification. If this were the case, the consequence would be the need to build an in-home-made database with a larger number of mosquitoes.

The MALDI-TOF MS tool has proven its effectiveness in identifying individual blood meals. However, in the field it is important to be able to identify multiple blood meals, as some studies have shown that mixed blood meals can be found in up to 10% of mosquitoes collected [13, 15]. Multiple host feedings (i.e. single mosquitoes taking blood from different types of hosts) have been observed and described by other authors in the field of entomological surveys [2, 12]. The effectiveness of the MALDI-TOF MS method opens other perspectives such as the identification of interrupted blood meals.

## Competing interests

The authors declare that they have no competing interests.
